# Sentinel Node Biopsy for Breast Cancer Patients: Issues for Discussion and Our Practice

**DOI:** 10.4061/2011/109712

**Published:** 2010-12-28

**Authors:** Georgios Pechlivanides, Dorothy Vassilaros, Anastasios Tsimpanis, Anastasia Apostolopoulou, Stamatis Vasilaros

**Affiliations:** “Prolipsis” Diagnostic Center, Breast Unit, 88A Mihalacopoulou Street, 11528 Athens, Greece

## Abstract

Sentinel node biopsy has been established for several years now as a standard procedure of breast cancer surgery, but there are several variations of the indications and the technique used. This paper provides information regarding several issues of debate for its application as are the selection criteria, the application to patients with multifocal/multicentric breast cancer or DCIS, postneoadjuvant chemotherapy, the necessary number of nodes to be biopsied, the need for lymphoscintigraphy, the technique for frozen section, the factors that may predict nonsentinel nodes (NSNs) involvement, the value of micrometastasis and isolated tumour cells, the internal mammary chain sentinel nodes, and finally the axillary recurrence after SLNB. Our view for these issues is included together with our experience of 430 SLNBs.

## 1. Introduction

Lymph node status is a key factor in determining the stage of breast cancer and the most appropriate therapy and for predicting the outcome of patients. Accurate identification of sentinel lymph nodes (SLNs) preoperatively is of clinical importance. The results of the NSABP B-32 study indicate the superiority of the SLNB compared to the ALND treatment approach relative to postsurgical morbidity outcomes over a 3-year follow-up period [1]. Also the use of ipsilateral upper arm is not restricted if only SLNB is applied. In the sentinel lymph node (SLN) era, axillary lymph node dissection (ALND) for uninvolved axillary lymph nodes should be considered unnecessary and inappropriate. The sentinel lymph node biopsy sensitivity is more than 90%, its specificity 100%, its accuracy more than 95%, and the axillary recurrence rate is less than 1%. There are still though some disputable issues also on this subject.

## 2. Selection Criteria for a SLN Biopsy

ASCO Expert Panel published in 2005 guidelines in which SLNB was not acceptable for T3 or T4 tumors, inflammatory breast cancer, ductal carcinoma in situ (DCIS) without mastectomy, nodes suspicious for metastasis, pregnancy, prior axillary surgery, prior nononcologic breast surgery, and after preoperative systemic therapy [2]. Is it possible though to predict to which patients we should not perform the procedure? According to Lynch et al. [3] there are* clinical and pathologic features which are associated with a positive SLN,* such as a palpable tumor, increasing tumor size, increasing histologic grade, and angiolymphatic invasion. Some of the above features are unreliable and for other we do not have information always. 

Regarding *tumor size*, SLNB may be acceptable also for patients with T3 or T4b tumors according to Takei et al. [4], even though SLN identification is lower. Yet SLN involvement is higher compared with T1 or T2 tumors, and systemic adjuvant therapy is obviously needed for patients with T3 or T4b tumors. SLNB is only a bridge to further axillary treatment such as ALND or axillary RT for those patients. *Clinical examination of the axilla* is always the first approach to select the candidate for SLNB. This approach though, is inaccurate in 41% of cases, false positive in 53% of patients with moderately suspicious nodes and 23% of patients with highly suspicious nodes. False positive results are less frequent with larger tumor size and higher histological grade, but are not associated with age, body mass index, or previous surgical biopsy [5]. Nodes clinically suspicious for metastasis should not be considered a contraindication to SLNB, since palpable axillary lymph nodes can be identified and removed by SLNB [4]. It has been recently shown that by applying the procedure also to patients with clinically suspicious nodes, after neoadjuvant treatment, large tumors >2 cm, multifocal disease, and previous excisional biopsy the number of unnecessary ALNDs has been decreased from 26% to 9% [6].

One would consider that there is not a discrete borderline for the selection criteria and this may be anywhere between to perform SLNB to “all patients” and to perform it to “only small tumors with ultrasound guided FNA negative axillary nodes”.

In our unit candidates for SLNB are all patients with tumor diameter of less than 3 cm, and with negative axilla both clinically and ultrasonographically. With these selection criteria we have performed 430 SLNBs. The size of less than 3 cm criterion is not supported by the literature, on the contrary. Being very conservative, we have only recently moved to apply SLNB in all T2 tumours.

## 3. Multifocal (MF) or Multicentric (MC) Breast Cancer

In these cases there is an issue when we consider the diameter of the tumor in relation to the possibility of lymph node metastasis. Tresserra et al. [7] found that lymph node metastasis is related only to the diameter of the largest tumor. Ferrari et al. showed that in 93.3% of patients with multifocal or multicentric cancer the lymphatic pathways from two different sites of injection converged into one major trunk leading to the same SLN(s) and in 6.7%, mainly multicentric cancer, two different pathways found each of them leading to a different SLN [8]. The false negative rate was 7.1% in this study and the authors suggested that both MF and MC tumors do not represent a contraindication for SLNB. On the contrary a French prospective multi-institutional study found that the false negative rate of SLNB for multiple unilateral synchronous breast cancer was 13.6% which is unacceptably high even for small tumors [9]_._ Regarding MC patients it is generally recommended not to perform SLNB. Multifocality and multicentricity consist a contraindication for SLNB in our unit because we aim for a false negative rate of less than 5.9%. This was achieved during the testing period of the first 100 procedures. Multifocal tumours recently are treated differently, as we perform SLNB for the largest focus on a trial's base. 

## 4. Avoiding SLNB

The most reliable so far method to detect involved axillary nodes is *ultrasound and FNA *of the suspicious on palpation and/or ultrasound nodes. In the study of Deurloo et al. this procedure can find 31% of all tumor-positive axillae (macro + micrometastases) and 41% of all axillae containing macrometastases with maximum cortex thickness being the main feature to predict metastatic involvement [10]. Markedly hypoechoic, thick, or lobulated cortex and eccentric or absence of fatty hillum are the malignant features according to Koelliker et al. [11]. Jung et al. found the sensitivity, specificity, and positive and negative predictive values of the ultrasound alone of axillary LNs for metastatic breast cancer were 54, 91, 75, and 81%, retrospectively. For the US-FNAC, the respective values were 80, 98, 97, and 84% [12]. AUS with needle biopsy reduces the need for SLNB by 54% and affects treatment in patients with cT2 or greater breast cancer [13]. The absence of fatty hillum has the highest positive predictive value of 93% in the study of Garcia-Ortega et al. [14]. From the same study the sensitivity and specificity of axillary ultrasonography are 63.2% and 88.7%, respectively. The sensitivity and specificity of *axillary core biopsy* are 69.1% and 100%, respectively. With this procedure sentinel lymph node biopsy can be avoided in 33% of initial candidates and immediate breast reconstruction was undertaken in 35.1% of the patients with mastectomy and negative axillary core biopsy. Moreover breast cancer is frequently characterized by increased 2-fluoro-2-deoxy-D-glucose uptake, and many studies have shown encouraging results in detecting axillary lymph node metastases. The sensitivity of *FDG-PET scan for detection of axillary lymph node metastases* in the study of Veronesi et al. [15] was low (37%); however, specificity and positive predictive values were acceptable (96% and 88%, resp.). The high specificity of PET imaging indicates that patients who have a PET-positive axilla should have an ALND rather than an SNB for axillary staging. In contrast, FDG-PET showed poor sensitivity in the detection of axillary metastases, confirming the need for SNB in cases where PET is negative in the axilla. 

Axillary ultrasound has a sensitivity of around 50% and a specificity of more than 90% in our hands and therefore we rely only on the positive results.

## 5. Neoadjuvant Chemotherapy

So far SLNB is not acceptable for patients with positive nodes in the axilla at initial diagnosis even if their axillary metastases are downstaged to negative by neoadjuvant chemotherapy. In theory, lymphatic mapping may not accurately show whether nodal metastases exist after preoperative chemotherapy because of excessive fibrosis of the tumour lymphatics and/or the potential obstruction of lymphatic channels with cellular material or tumour emboli [16, 17]. Thus, it is important that the feasibility and reliability of SLNB is determined in this patient group. 

Three meta-analyses have been reported that examined the results of SLNB in patients with breast cancer who did not receive preoperative chemotherapy [18–20]. The overall sentinel lymph node identification rate (IR) calculated in the largest of these, determined from data on 28 studies, was 90 per cent. The estimated IR for SLNB after preoperative chemotherapy in the meta-analysis of Xing et al. [21] in which data from the 21 studies were analyzed was 91 per cent. The fact that the rates of sentinel lymph node identification were similar in pooled analyses suggests that the concerns mentioned previously [16, 17] are not serious. The estimated sensitivity of SLNB after preoperative chemotherapy was 88 (95 per cent, credible interval 84 to 91) per cent, with a false-negative rate of 12 per cent. The overall false negative rates for SLNB determined in the three separate meta-analyses of patients who did not receive preoperative chemotherapy were 8.4 per cent [22], 5.1 per cent [23], and 9 per cent [18]. It is still controversial whether SLNB is acceptable for patients with clinically positive nodes at initial diagnosis who are treated with neoadjuvant chemotherapy, whereas SLNB alone is acceptable for patients with an initial diagnosis of clinically negative axilla who are treated with neoadjuvant chemotherapy. SLN identification rate was 65% in patients with clinically positive nodes at initial diagnosis; however, it was 100% in patients with clinically negative presentation who were treated with neoadjuvant chemotherapy at M. D. Anderson Cancer Center [24]. On the other hand proponents of SLNB would argue that in women who have had preoperative chemotherapy the clinical impact of understaging is less significant, given that they were assigned a clinical stage before chemotherapy and so the decision to give systemic therapy had already been made. Thus SLNB after chemotherapy provides information about residual nodal disease and guides regional therapy. There is also the consideration of NAC downstaging the axilla, converting N1-N2 lymph node status to N0 and also avoiding full axillary dissection in these patients, provided that the false negative rate is low as it was found to be 4.3% in the study of Schwartz et al. [25].

Shimazu et al. [26] proved that intraoperative frozen section (FS) analysis of SLNs is as accurate for neoadjuvant chemotherapy-(NAC-)treated as for non-NAC-treated patients, which indicates that FS analysis of SLNs is a clinically acceptable method for those receiving NAC. The application of SLNB- to NAC- treated patients has been proved to have similar sensitivity, specificity, and accuracy to non-NAC-treated patients (74, 100, and 88%, versus 71, 99, and 90%). The sensitivity of FS analysis for macrometastases is lower for NAC-treated patients (76%) than for non-NAC-treated patients (91%), while that for micrometastases and isolated tumor cells is higher for NAC-treated patients (67%) than for non-NAC-treated patients (31%). However, neither of these differences is statistically significant. 

Neoadjuvant chemotherapy can be given not only to patients with locally advanced breast cancer, but also to those with axillary lymph node metastasis and an operable tumor for down-staging and to downsize a tumor in order to perform conservative surgery. However, SLNB after NAC results in a lower identification rate and a higher FNR than SLNB before treatment. Recently, a hybrid imaging device has been developed, which consists of single photon emission computed tomography (CT, SPECT) and a low-dose CT installed on the same platform. This imaging system offers an easy and safe method of performing SLNB under local anesthesia. To identify the initial cancer stage in patients who will be treated by systemic therapy before surgery, SLNB should be performed prior to systemic treatments, according to Iwase et al. [27] by using a well-developed navigating tool, such as SPECT/CT or the radioguided. 

Neoadjuvant chemotherapy or hormone therapy for operable cancer is still under investigation in our unit and axillary clearance is a standard procedure for these patients.

## 6. DCIS

DCIS is pathologically diagnosed only after complete removal of the tumor, and the incidence of accompanying microinvasion increases when the tumor is palpable and large, is of high grade, or if the patient is young [28]. The ASCO guidelines showed a cutoff diameter of 5 mm or larger, for which SLNB is recommended for patients with an initial diagnosis of DCIS [2]. In addition, SLNB is recommended for patients who will undergo mastectomy for the treatment of DCIS, because the ability to perform SLNB is lost after removal of the breast. 

Our standard procedure is to be prepared and perform SLNB (radioguided or with blue dye) when frozen section results show DCIS. We prefer to exhaust our efforts not to need a second operation, which is necessary in almost 15% of cases where an infiltration or microinfiltration is found on final histology.

## 7. Lymphoscintigraphy

The need to perform lymphoscintigraphy prior to SLNB is another issue. It has been proved beneficial in showing that at least 1 radioactive SLN will be identified intraoperatively, but it does not accurately predict the number of SLN in 40–50% of the patients [29, 30]. The number of hot spots in preoperative mapping should serve as a rough indicator of the smallest number of nodes the surgeon should attempt to resect, but not the exact number of nodes expected to be found.

Since we do not do internal mammary chain SLNB, we find lymphoscintigraphy not helpful from the surgical point of view except in cases with a history of prior sentinel node, axillary dissection, and plastic surgery. Nevertheless our nuclear medicine department finds its images reassuring for the efficacy of their job.

## 8. Number of Sentinel Nodes

The improvement of experience with the blue dye procedure along with the addition of radioisotope marking of the SLN contributed to the increase of the number of SLN are biopsied. Palpable tumors, surgeon's inexperience, and dermal injection are associated with greater than 4 SLNs identified. All 3 of these factors remain significant on multivariate analysis [31]. Low and littlejohn [32] suggest that the optimal number of SLN to harvest, after intradermal injection of both isotope and blue dye, is two. In their study 33 patients had positive SLN results. If only the first SLN was analyzed, 87.9% of those positive biopsies would have been discovered. Two SLNs raised the predictive value to 97.0%. Lynch et al. [3] identified a mean number of 2.86 (range, 1–8) SLNs after periareolar injection of radiolabeled technetium sulfur colloid on the day of surgery. Among the 38 patients with a positive SLN (30.2%), the hottest node was the first positive SLN in 27 patients (71.1%). The first positive SLN was the first node removed in 31 patients (81.6%) and the second node in 37 patients (97.4%); it was removed in all patients by the third SLN. These data support the trend of limiting SLN biopsy to 3 lymph nodes. Removing all SLNs with radioactive counts greater than 10% of the ex vivo counts of the hottest SLN does not increase accuracy. The false negative rates were 14.3% and 4.3% for patients with a single sentinel node versus multiple sentinel nodes removed, respectively, in the study of Wong et al. [33]. The blue dye injection alone was the only factor independently associated with identification of a single SLN and patient age, tumor size, tumor location, surgeon's previous experience, and type of operation were not significant.

It is also our finding that blue dye staining only leads in most cases to a single SLNB. Our average SLN number is 1.9 by following the rule of 10%.

## 9. Factors That Predict Nonsentinel Nodes (NSNs) Involvement

It is accepted in the community that a positive SLN frozen section should be followed by ALND. Attempts have been made to identify factors that predict non sentinel nodes (NSNs) involvement. The rate of NSNs involvement increases proportionately to the size of both SN metastases and primary tumor, while no significant correlation was found for lymphovascular invasion. At univariate and multivariate analysis of findings from cases with multiple probe-detected hot nodes, positivity in more than one hot node is the strongest predictor of NSN involvement [34]. More than one positive SLN and a ratio of positive SLNs to total SLNs of greater than 0.5 were found by Tan et al. [35] to be predictors for additional axillary nodal involvement in both univariate and multivariate analyses. The number of positive SLNs and the ratio of positive SLNs to total SLNs is an indication of total tumor burden in the sentinel nodes and may be a reflection of the propensity of the tumor for further lymphatic invasion in the axillary basin. Another assumption made is that some patients may benefit from a more conservative surgical approach to their axillae, perhaps limited to sentinel node biopsy only or to axillary procedures restricted to the group of axillary nodes in close proximity to those designated as sentinel nodes. This assumption was made when Samoilova et al. [36] found that all patients with sentinel node tumor deposits < or =5 mm had three or fewer positive nodes; 95% were sentinel node-positive only, and 91% had single-node involvement. Nine models have been developed until now to predict non SN status in patients with SN metastasis. Four models are nomograms: the Memorial Sloan-Kettering Cancer Center nomogram (MSKCC nomogram), the Mayo nomogram, the Cambridge nomogram, and the Stanford nomogram. Three models are scoring systems: the Tenon score, the score from the M.D. Anderson Cancer Center (MDA score), and the score of the group of Saidi. Finally, two are recursive partitioning tools developed by the group of Kohrt. Those models have been compared by Coutent et al. [37]. They found that all models do not perform equally, especially for the subgroup of patients with only micrometastasis or ITC in the SN overall, the MSKCC nomogram and Tenon score outperform other methods for all patients, including the subgroup of patients with only SN micrometastases or ITC, but need extensive testing before they are put into clinical practice. A new perspective of non SLN metastasis prediction is the presence of extracapsular invasion of the SLN and it was studied by Fujii et al. [38]. It seems to be a strong predictor of residual disease in the axilla. All cases of positive nodes in NSLN in these series had extracapsular invasion at the metastatic SLNs. Furthermore, the absence of ECI of SLN was significantly associated with the absence of metastasis in the NSLN (*P* < .001).

Our experience with 12.5% chance of other non-SLN infiltrated on ALND when one only SLN is infiltrated does not allow us not to proceed to ALND in these circumstances. The case of only one SLN with microinfiltration is still under investigation because of the small number of patients.

## 10. Intraoperative Assessment

The need for intraoperative assessment of the SLN is not under discussion any more as it saves the patient from a second operation most of the time. False negative rate of frozen section is found to be 5–25% percent (not surprisingly greater for micrometastases) and a second operation cannot be avoided always. Its sensitivity may be improved by multilevel sectioning of the lymph node and by histochemistry [39–41]. 

*Imprint cytology* has been tried and is still practiced, but failed to achieve results similar to frozen section. The meta-analysis of 31 studies published in 2005 showed that pooled sensitivity of imprint cytology was 63% and specificity was 99%. Pooled sensitivity for macrometastases was 81% and that for micrometastases 22%. Frozen sectioning had better sensitivity than imprint cytology in three of four direct comparisons [42]. More recent studies comparing frozen section and rapid immunohistochemistry to touch imprint cytology did not change these findings [43]. 

*Ultrarapid cytokeratin IHC* assay is a procedure that does not exceed 20 min. Compared to *frozen hematoxylin-eosin (H&E) stain* has a sensitivity of 85% versus 70%, a specificity of 100% for both and accuracy rate of 96% versus 92%, respectively [44]. Ultrarapid IHC may detect also sentinel node micrometastasis and isolated tumor cells (ITCs) [44–46]. Serial sections with a spacing of 150 microns between following sections seems to increase the ability of IHC to detect ITCs [46]. *One Step Nucleic Amplification *which is a method that amplifies cytokeratin 19 mRNA and measures its amount which is directly related to the size of metastatic foci. This is a procedure that is completed in 30 min. In a multicentric study in Japan it was found that its concordance rate to the histochemical investigation is 98.2% the specificity is 96.5% [47]. Concerns are raised though for the inability to determine the actual size of nodal metastases which is important for therapeutic decisions and the inability to determine the true false positive and negative rate, since the tissue has been used for RNA isolation. 

Frozen section with ultra rapid cytokeratin IHC is the way we proceed. In our series SLN was found negative on H&E and positive on IHC in 3.17% of patients and the discrepancy between H&E and IHC was significantly less common when more than one SLN were examined (1.6% versus 3.7%, *P* < .01).

## 11. Micrometastasis and Isolated Tumor Cells

The importance of detecting micrometastasis (MM) (0.2 mm–2 mm) and isolated tumor cells (ITCs) (<0.2 mm) in an ALN is unknown. Should its detection in a SLN on ultra rapid IHC lead to ALND? In the study of Dabbs et al. [48] 13.6% of the patients that were IHC positive had ALN macrometastasis in a solitary ALN. Of the patients with micrometastatic SLNs 8.1% had a solitary positive ALN, 6.1% of which were macrometastases. Overall 9.0% with traditionally defined SLN micrometastases of 2.0 mm or less had a solitary ALN macrometastasis. There was a significant difference in the means of SLN tumor sizes for the SLN-positive/ALND-negative (4.5 mm) versus SLN-positive/ALND-positive (10.1 mm) patients. When the 2 recently published interpretations of the TNM definitions were applied to cases of low-volume sentinel lymph node (SLN) involvement and their corresponding non-SLNs for reclassification as micrometastasis or ITC, the rates of non-SLN metastases associated with SLN ITCs were 8.5% and 13.5%, respectively [49]. The prognostic impact of micrometastases and ITCs is still under investigation. Isolated tumour cells or micrometastases in regional lymph nodes were associated with a reduced 5-year rate of disease-free survival among women with favourable early-stage breast cancer who did not receive adjuvant therapy in the study of de Boer et al. In patients with isolated tumour cells or micrometastases who received adjuvant therapy, disease-free survival was improved [50]. Ten-year breast cancer-specific survival (BCSS) and overall survival (OS) in pNmic breast cancer were found by Truong et al. to be significantly lower compared to pN0 disease (BCSS 82.3% versus 91.9%, *P* < .001 and OS 68.1% versus 75.7%, *P* < .001) [51]. Park et al. showed that ITC have no impact on survival at a median 8.2 years of followup, whereas MM shows a trend toward poorer disease-free survival (DFS) (*P* = .091, log rank) and distant disease free survival (DDFS) (*P* = .066) and significantly reduced BCSS (*P* = .016). In multivariate analyses, detection of MM is an independent prognostic factor for DDFS (*P* = .025) and BCSS (*P* = .01) in adjuvant untreated patients. The evidence so far shows that micrometastases in axillary lymph nodes have prognostic impact. This is not found for ITC. Those findings support the use of systemic adjuvant therapy in patients with MM [52]. Axillary recurrence could also be a threat for those patients, and it was found that one patient with an SLN micrometastasis (1 of 33; 3%) and 1 patient with an SLN macrometastasis (1 of 14; 7%) developed an axillary recurrence with distant metastasis at 84 months and 28 months, respectively [53]. The group from MSKCC found that young age, estrogen receptor negative status, high MSKCC nomogram score, and chemotherapy were associated with ALND. The practice of selectively limiting ALND to IHC-only patients thought to be at high risk and to patients for whom the identification of additional positive nodes may change systemic therapy recommendations seems to be a safe and reasonable approach. Among patients who had ALND (*n* = 95), 18% had a positive non-SLN. No axillary recurrences were observed in this series with a median followup of 6.4 years. The percentage of patients who were recurrence-free after 5 years was 97 [54]. 

There is not though a general agreement for the proper way of sentinel node specimen handling in order to achieve finding all MM and ITCs. There are institutions doubting the necessity of multiple level sectioning [55] and other supporting the Milan proposal of sectioning at 50-micron intervals and for each level, one section stained with hematoxylin and eosin and the other section immunostained for cytokeratins using a rapid immunocytochemical assay, claiming that this way the detection of metastases is increased by 7.8% [56–58]. American College of Pathologists guidelines of 2009 are that the SN should be bivalved along the longitudinal axis, serially sectioned at 1.5- to 2.0-mm thickness blocks; each block should be sectioned at 3 levels and examined using routine H&E stains. They consider controversial the routine use of immunohistochemical (IHC) staining or other molecular approaches. 

We perform ALND for all patients with MM. We do not proceed to ALND for patients with ITC if it is the only positive node among 2 or more SLN. If MM is found on regular histology we discuss with the patient the options of doing nothing or having ALND or axillary RT, informing her also of the existing risk of leaving an infiltrated node in her axilla.

## 12. Internal Mammary Chain Sentinel Nodes

Lymphatic mapping for sentinel lymph node (SLN) biopsy has demonstrated extra-axillary drainage in up to 35% of patients. In the subset of patients with tumours 1 cm or less in size and no ALNM, information on IMN status would provide important information. In these cases, the presence of IMN metastases would change the staging from stage I to stage IIIB, according to the current tumour, node, and metastasis classification. More importantly, it would influence these patients' adjuvant treatment [59]. Peritumoral isotope injection contributes to internal mammary chain (IMC) sentinel lymph nodes visualization in 28.75% of patients according to Bourre et al. [60]. IMC biopsy failed in 4% of patients. IMC sentinel node was infiltrated in 4.8% of biopsies performed. Prophylactic irradiation of the IMC was indicated in 376 patients. Therefore such information should make it possible to personalize treatment for patients with stage cT1 mammary cancer and thereby avoid needless internal mammary radiation therapy in a large number of patients (93.4% in this study). By intratumoral isotope injection and blue dye injection the IMC sentinel node was visualized in 21.5% of patients and could be harvested in 87% from the study of Estourgie [61]. IMC SLN contained tumor in 17% of those harvested and in 7% IMC nodes were positive whereas the axilla was tumor free. There was a change of management in 29% of the patients with a successful IMC-SLNB, including institution or omission of radiotherapy to the IMC, adjuvant systemic therapy, or omission of the axillary node dissection. In the European Institute of Oncology study IMC nodes were found in 88% of patients, and 8.8% were positive which modified the radiotherapy and systemic treatment [62].

IMC nodes have only lately been a subject of reconsideration in our unit and the same is the site of radiotracer injection as by the subdermal injection we use IMC nodes that are not visualized. For the time being we perform a biopsy from the IMC LNs close to the inner site tumours.

## 13. Axillary Recurrence after SLNB

The incidence of axillary recurrence after tumor negative sentinel node biopsy, in the study of Bulte et al. [63], is 0.6% (3/541). An event occurred in the 11% of patients with a micrometastasis in the sentinel node. This was not significantly different from the patients with a tumour-free sentinel node. In the same study was observed a non-significant different risk of distant disease in case of micrometastases compared to a tumour negative sentinel node. Also the accuracy of SLNB in multicentric/multifocal breast cancer was comparable with that observed in unifocal breast cancer with low false negative rate and no axillary recurrence in the study of Holwitt et al. [64]. Despite a lower rate of SLN positivity in patients undergoing SLNB only, axillary recurrence was not observed and none of the 52 patients experienced axillary recurrence (median followup 4.8 years). In 2008 a meta-analysis was published of 48 selected studies concerning 14959 sentinel node-negative breast cancer patients followed for a median of 34 months [65]. Sixty-seven patients developed an axillary recurrence, resulting in a recurrence rate of 0.3%. The sensitivity of the sentinel node biopsy was 100%. Uni- and multivariable variable analyses showed that the lowest recurrence rates were reported in studies performed in cancer centers, in studies that described the use of (99 m)Tc-sulphur colloid, and also when investigators used the superficial injection technique or evaluated the harvested sentinel nodes with haematoxylin-eosin and immunohistochemistry staining (*P* < .01). These results suggest that the sentinel lymph node procedure is a reliable and accurate instrument for staging of patients with early breast cancer.

Following the criteria we have set our axillary recurrence rate is 0.3% and this happened to one patient who did not complete chemotherapy.

## 14. Conclusions

Most of the above discussed issues are still in debate. Large tumour size and multifocality not contraindication for SLNB if we accept a slightly lower identification and increased false negative rate. Axillary ultrasound with FNA or core biopsy is accepted as helpful, because of its high specificity, in order to decrease the number of SLNBs. The role of SLNB in relation to neoadjuvant chemotherapy is still in debate and the same applies to DCIS. Lymphoscintigraphy is not helpful. Two or three are the optimum number of SLNs to be biopsied. There are no widely accepted rules to predict the non-SLN metastasis. Frozen section with ultra rapid cytokeratin assay is the most preferred procedure for its sensitivity in defining lymph node micrometastasis, which is related to poorer prognosis. Internal mammary chain SLNB may change the management in few patients. The axillary recurrence rate with SLNB is acceptably low and this allows us to try and expand its indications.
